# Prevalence of loneliness among older adults in Germany

**DOI:** 10.25646/11664

**Published:** 2023-09-20

**Authors:** Susanne Wurm, Ulrike Ehrlich, Frauke Meyer-Wyk, Svenja M. Spuling

**Affiliations:** 1 University Medicine Greifswald, Department Prevention Research and Social Medicine, Germany; 2 German Centre of Gerontology, Berlin, Germany; 3 European Commission, Joint Research Centre (JRC), Ispra, Italy

**Keywords:** LONELINESS, SOCIAL NETWORK, SOCIAL RELATIONSHIPS, GERMAN AGEING SURVEY 2020/2021, AGING, GERMANY

## Abstract

**Background:**

Loneliness refers to the subjective perception of a mismatch between a person’s social needs and their actual personal relationships. In this paper, the prevalence of loneliness in the older population was examined based on current data.

**Methods:**

The German Ageing Survey is an ongoing, population-representative study. A total of 4,261 people 50 years of age and older were surveyed in 2020/2021 with regard to their experience of loneliness.

**Results:**

Overall, 8.3 % of the population 50 years of age and older feel lonely. The findings showed no differences between different age groups over 50 years of age, nor are there gender or educational differences.

**Conclusions:**

There was no evidence that older individuals living in private households experience loneliness more commonly than middle-aged individuals. Data from nursing home residents indicate that there may be a higher risk of loneliness.

## Introduction

Loneliness describes the perceived gap between a person’s social needs and their actual personal relationships, both in terms of quantity and quality of the relationships [1]. Loneliness is a subjective feeling. Therefore, people can feel lonely despite having a large social network and, conversely, objectively socially isolated people do not necessarily feel lonely [2].

Loneliness is associated with a health risk. Several studies have shown associations between loneliness and a higher risk of cardiovascular disease, depression, cognitive impairment and Alzheimer’s dementia [2, 3]. In addition, loneliness is associated with increased use of physicians and a higher likelihood of premature death (e.g. [2, 4]).

Previous findings on the risk of loneliness in different population groups indicated that the probability of feeling lonely does not increase across age groups. According to results of the German Ageing Survey (DEAS) as well as an international meta-analysis, the risk of loneliness at an older age is not higher than in the middle of adulthood [5–8]. Studies on the very-old population in Germany complement the findings of the DEAS. They show for the group of 80-year-olds and older that about every 17th to 20th person (5–6 %) is lonely at this age [9, 10]. These findings also demonstrate that loneliness is not more widespread among old-age people than among younger age groups. Differences in the figures on the prevalence of loneliness are partly due to the fact that loneliness was measured differently depending on the study, and people living in nursing homes were sometimes not surveyed. While social networks are often smaller at an older age than in younger age groups [11], loneliness is not more prevalent. Some studies indicated that women and men do not differ in their risk of loneliness starting from middle adulthood and onwards [5], while among children, adolescents and young adults, slightly higher loneliness risks are detected among boys and men [12].

There are also varying and sometimes contradictory results with regard to the educational status. For example, around 7 % of highly educated people reported feeling lonely in the DEAS compared to almost 15 % of those with low levels of education [5].

Based on nationally representative data from the DEAS, the present study examined the current prevalence of loneliness among women and men, in various age and education groups among the population 50 years of age and older. In addition, the study investigated how the rate of people being at risk of loneliness has changed compared to the pre-pandemic period.

## Indicator

The loneliness rate in the German Ageing Survey (DEAS) 2020/2021 is captured on the basis of an indicator through the self-reports of the respondents in a questionnaire that was completed in written form or online. The DEAS is a nationwide representative cross-sectional and longitudinal survey of people who are in the second half of life and are thus at least 40 years of age. The first survey took place in 1996, and six follow-ups have taken place since then.

In the 2020/2021 survey year, 5,402 people between the ages of 46 and 100 participated in the oral interview; 4,419 of these respondents (82 %) also completed the additional questionnaire. The internationally established loneliness measure (LONE scale [1]) used in the questionnaire, which is based on a total of six statements, contains statements such as ‘I miss having people around among whom I feel comfortable’ or ‘I miss emotional security and warmth’. Affirmation of these statements can be expressed on a scale from 1 ‘strongly disagree’ to 4 ‘strongly agree’. People are classified as lonely if their individual scale mean was greater than 2.5 in the possible value range from 1 to 4. Respondents with missing data were excluded (21 respondents: 11 women, 10 men). In addition, respondents who were younger than 50 years of age (106 respondents: 56 women, 50 men) or older than 90 years of age were excluded (31 respondents: 11 women, 20 men). The final analytical sample consisted of 4,261 respondents between 50 and 90 years of age (2,179 women, 2,082 men).

The 1997 International Standard Classification of Education (ISCED) was used to classify respondents’ educational and vocational qualifications [13]. Weighted prevalences as percentages with 95 % confidence intervals (95 % CI) were presented on loneliness stratified by gender, age and education using methodology that takes into account the stratified sampling of the DEAS. Descriptive results with the respective confidence intervals are presented in tabular form. In addition, a significance test was conducted to test for differences between the groups. A detailed description of the DEAS methodology is presented elsewhere [14, 15].

## Results and conclusion

A total of 7.8 % of women and 8.8 % of men in Germany reported feeling lonely in 2020/2021. There was no statistically significant difference between the loneliness rates of women and men. The proportion of women and men who were classified as being lonely was thus at a comparable level (Table 1). Moreover, there was no age-associated trend among the respondents, as there are no statistically significant differences between the age groups. Thus, the loneliness rates were at a comparable level in all age groups. Furthermore, the loneliness rate also did not show a statistically significant difference between different groups of education (Table 1).

The results show that loneliness does not vary between the age groups considered. In contrast, data from a comprehensive UK study indicate that the prevalence of loneliness may be higher among young adults than among older adults [16]. During the COVID-19 pandemic, loneliness increased in the general population, but there was no additional increase in loneliness among older adults [8, 16, 17]. Additional analyses based on the DEAS demonstrated that, as early as in the winter of 2020/2021, the loneliness rate had declined to a level comparable to 2017. The widespread notion that older people in particular are subject to loneliness is therefore not corroborated on the basis of the present results. It should be noted that the DEAS does not survey people living in nursing homes. A survey of around 1,000 people 80 years of age and older who live in a nursing home in Germany showed that more than one in three people feels lonely (35 %) and thus the proportion of lonely people in nursing homes is significantly higher than in the general population [18]. Moreover, living alone should not be mixed up with feeling lonely. The rate of people living alone among the people over 85 years of age is 63 %, which is about the same level as among the under 25-year-olds [19].

An explanation for the finding that old age is not per se correlated to higher rates of loneliness is provided by the so-called socioemotional selectivity theory [20]. According to this theory, social needs change with age, so that a smaller number of close relationships is perceived as emotionally satisfactory in old age.

## Key statement

- 8.3% of the population 50 years of age and older reported feeling lonely in 2020/2021.
- Loneliness rates were at comparable levels for women and men 50 years of age and older. They do not depend on education.

## Figures and Tables

**Table 1:** Loneliness rate by gender, age and education (n = 2,179 women, n = 2,082 men) Source: German Ageing Survey (2020/2021)

	%	(95 % CI)
**Total (women and men)**	**8.3**	**(6.5 – 10.5)**
**Gender**		
Female	7.8	(5.5 – 11.0)
Male	8.8	(6.3 – 12.1)
**Age group**		
50 – 64 years	9.6	(6.7 – 13.2)
65 – 74 years	6.1	(4.2 – 8.9)
≥ 75 years	7.6	(4.4 – 12.9)
**Education group**		
Low/medium	8.2	(6.1 – 10.9)
High	8.5	(5.8 – 12.5)

CI = confidence interval
